# Kin Recognition in an Herbicide-Resistant Barnyardgrass (*Echinochloa crus-galli* L.) Biotype

**DOI:** 10.3390/plants12071498

**Published:** 2023-03-29

**Authors:** Le Ding, Huan-Huan Zhao, Hong-Yu Li, Xue-Fang Yang, Chui-Hua Kong

**Affiliations:** 1 College of Resources and Environmental Sciences, China Agricultural University, Beijing 100193, China; s20223030381@cau.edu.cn (L.D.); lihongyulq2011@163.com (H.-Y.L.); 2 College of Geography and Environmental Science, Henan University, Kaifeng 475004, China; zhaohh@henu.edu.cn; 3 College of Life Science, Hebei University, Baoding 071000, China; yang_xue_fang1@126.com

**Keywords:** allelopathy, biomass allocation, flowering and reproduction, herbicide resistance, kin recognition, (–)-loliolide, rice–barnyardgrass interactions, root behavior

## Abstract

Despite increasing evidence of kin recognition in natural and crop plants, there is a lack of knowledge of kin recognition in herbicide-resistant weeds that are escalating in cropping systems. Here, we identified a penoxsulam-resistant barnyardgrass biotype with the ability for kin recognition from two biotypes of penoxsulam-susceptible barnyardgrass and normal barnyardgrass at different levels of relatedness. When grown with closely related penoxsulam-susceptible barnyardgrass, penoxsulam-resistant barnyardgrass reduced root growth and distribution, lowering belowground competition, and advanced flowering and increased seed production, enhancing reproductive effectiveness. However, such kin recognition responses were not occurred in the presence of distantly related normal barnyardgrass. Root segregation, soil activated carbon amendment, and root exudates incubation indicated chemically-mediated kin recognition among barnyardgrass biotypes. Interestingly, penoxsulam-resistant barnyardgrass significantly reduced a putative signaling (–)-loliolide production in the presence of closely related biotype but increased production when growing with distantly related biotype and more distantly related interspecific allelopathic rice cultivar. Importantly, genetically identical penoxsulam-resistant and -susceptible barnyardgrass biotypes synergistically interact to influence the action of allelopathic rice cultivar. Therefore, kin recognition in plants could also occur at the herbicide-resistant barnyardgrass biotype level, and intraspecific kin recognition may facilitate cooperation between genetically related biotypes to compete with interspecific rice, offering many potential implications and applications in paddy systems.

## 1. Introduction

Two or more plants occur together, resulting in a series of intraspecific and interspecific interactions. Plant–plant interactions, either positive or negative, are important driving forces for plant coexistence and community assembly [1,2,3]. Plant–plant negative interactions mainly include competition and allelopathy that are well-known in natural and managed ecosystems [4,5,6,7]. Positive or beneficial interactions involve in plant facilitation and kin recognition [8,9,10]. In contrast to competition and allelopathy, plant facilitation and kin recognition within and among species have received less attention, particularly for kin recognition [11,12]. Kin recognition allows plants to assess kinship to discriminate neighboring kin (collaborators) from non-kin (competitors), and then plants display morphological and biochemical plasticity toward reduced competition and defense when growing with kin, leading to increased fitness in kin groups [13,14]. Therefore, plant–plant beneficial interactions can result from kin recognition.

Kin recognition is a cooperative behavior among plants acting exclusively at the intraspecific level [15,16]. The ability to recognize and respond appropriately to close relatives may allow plants to tailor response strategies and optimize their competitive traits. Such kin recognition and cooperation have been observed at the individual, population, accession and cultivar levels in natural and cropping systems [17,18,19,20,21,22]. In natural systems, kin recognition in plants has dealt with conspecific individuals. Different from naturally occurring species where kin represent plants sharing the same mother, as either full or half siblings [17,23,24], in cropping systems, kin recognition occurs at the cultivar level because artificial selection generates crop cultivars with genetical and morphological uniform. Therefore, kin in crop plants are across closely related cultivars rather than siblings within a natural species [21,22].

Rice (*Oryza sativa* L.) is one of the principal grain crops. Barnyardgrass (*Echinochloa crus-galli* L.) is an intractable weed that coexists with rice in paddy systems for millennia. Rice–barnyardgrass represents a well-characterized model system to understand the combined roles of plant–plant intraspecific and interspecific interactions. However, barnyardgrass has evolved several herbicide-resistant biotypes duo to continuous use of herbicides in the past decades [25,26,27]. The incidence of herbicide-resistant barnyardgrass is escalating in rice fields. Accordingly, rice-barnyardgrass interactions should be altered by herbicide-resistant barnyardgrass biotype. Rice has to co-occur and interact with herbicide-resistant and -susceptible barnyardgrass, as well as normal barnyardgrass biotypes in paddies. However, the local coexistence and interactions among these biotypes are largely unknown. In fact, herbicide-resistant and -susceptible barnyardgrass usually come from a population with the same genetic background [28]. It is thought that kin recognition may occur between herbicide-resistant and -susceptible barnyardgrass biotypes.

Herbicide-resistant barnyardgrass has been a serious problem in paddy systems. Numerous studies have documented that allelopathic rice cultivars can release allelochemicals to suppress barnyardgrass and provide a competitive advantage for their own growth [29,30,31,32]. In particular, allelopathic rice cultivars interfered with both herbicide-susceptible and -resistant barnyardgrass while herbicide-resistant and -susceptible biotypes responded differently to allelopathic rice cultivars [28]. However, relatedness-mediated interactions between herbicide-resistant and -susceptible barnyardgrass, as well as their consequence for the interference of allelopathic rice cultivars with barnyardgrass biotypes retain obscure.

In the present study, we test whether kin recognition occurs at the herbicide-resistant barnyardgrass biotype level. To achieve this, we identified a penoxsulam-resistant barnyardgrass biotype with the ability for kin recognition from two biotypes of penoxsulam-susceptible barnyardgrass and normal barnyardgrass at different levels of relatedness. Furthermore, we determined the kin recognition responses of penoxsulam-resistant barnyardgrass with or without root segregation, soil activated carbon amendment and root exudates incubation. In addition, we examined the role of a putative root exudate signal in kin recognition among barnyardgrass biotypes. Finally, we assessed relatedness-mediated impacts on the interference of allelopathic rice cultivars with barnyardgrass biotypes. Together, these efforts provide a new insight into the kin recognition in plants, with a further understanding of rice-barnyardgrass interactions underlying the escalation of herbicide-resistant biotypes in paddy systems.

## 2. Results

### 2.1. Phenotypic Profile of Penoxsulam-Resistant Barnyardgrass in the Presence of Penoxsulam-Susceptible Barnyardgrass or Normal Barnyardgrass

**Figure 1:**
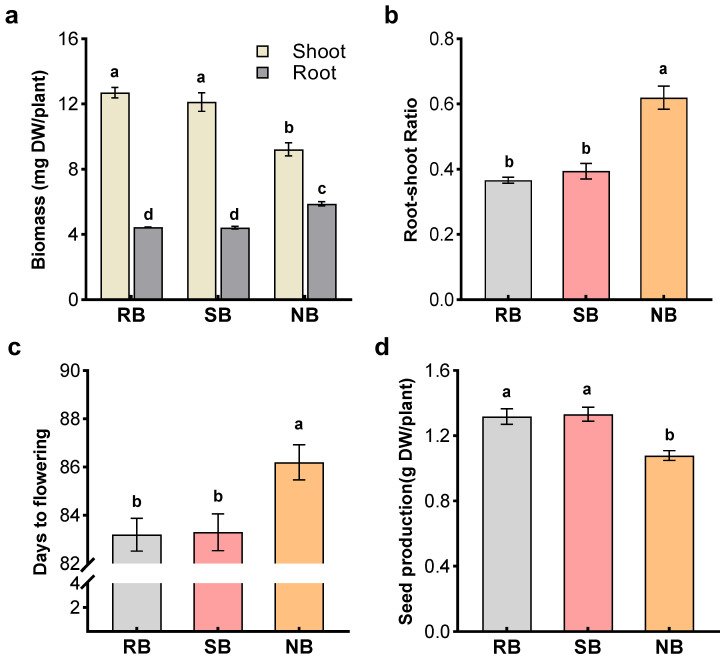
Biomass (**a**), Root-shoot radio (**b**), flowering time (**c**), and seed production (**d**) of penoxsulam-resistant barnyardgrass (**RB**) in the presence of penoxsulam-susceptible barnyardgrass (**SB**) and normal barnyardgrass (**NB**). Values plotted are means plus/minus SE. Columns with the same letter are not significantly different at *p* < 0.05 according to ANOVA, followed by Tukey HSD tests.

**Figure 2:**
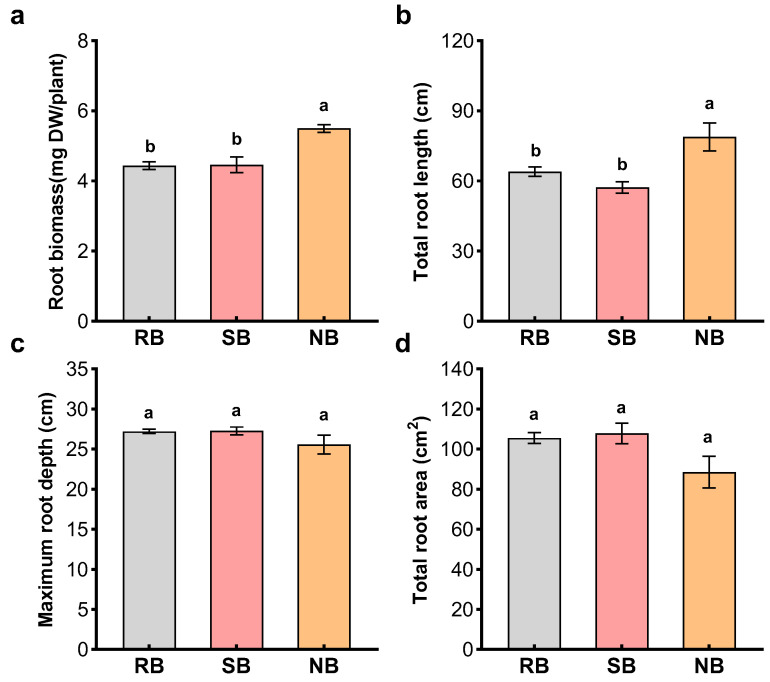
Root biomass (**a**), total root length (**b**), maximum root depth (**c**) and total root area (**d**) of penoxsulam-resistant barnyardgrass (**RB**) in the presence of penoxsulam-susceptible barnyardgrass (**SB**) and normal barnyardgrass (**NB**). Values plotted are means plus/minus SE. Columns with the same letter are not significantly different at *p* < 0.05 according to ANOVA, followed by Tukey HSD tests.

When penoxsulam-resistant barnyardgrass (focal biotype) was paired with itself, penoxsulam-susceptible barnyardgrass (closely related biotype, kin) or normal barnyardgrass (distantly related biotype, non-kin), phenotypic profile of penoxsulam-resistant barnyardgrass varied with neighbor relatedness (Figure 1 and Figure 2). There were not significant differences between penoxsulam-resistant and -susceptible barnyardgrass. However, the presence of distantly related barnyardgrass biotype significantly increased root biomass, reduced shoot biomass and seed production, and delayed flowering of penoxsulam-resistant barnyardgrass (Figure 1a–d). Similar changes with neighbor relatedness were observed in root measurements. Mixed-culture with the closely related penoxsulam-susceptible barnyardgrass did not alter the root measurements of penoxsulam-resistant barnyardgrass. However, distantly related barnyardgrass altered root measurements of penoxsulam-resistant barnyardgrass, and significant changes occurred in increased root biomass and total root length (Figure 2a,b). These results indicated that penoxsulam-resistant barnyardgrass adjusted phenotypic profile based on neighbor identity from different levels of relatedness, particularly for making plants with closely related biotype shifted biomass allocation from competitive roots to flowering and seed reproduction. Such relatedness-based phenotypic responses and biomass allocation indicated kin recognition in penoxsulam-resistant barnyardgrass biotype.

### 2.2. Chemically Mediated Kin Recognition in Barnyardgrass Biotypes

**Figure 3:**
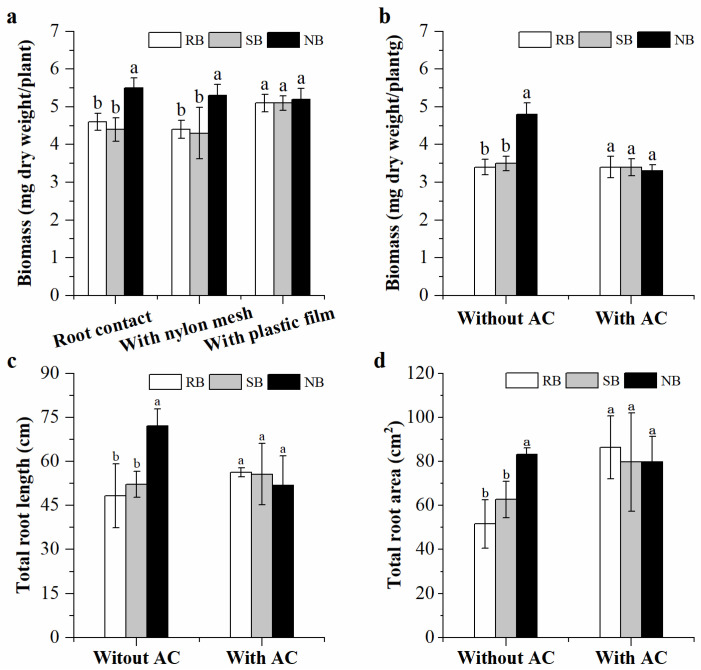
The effects of root segregation and soil activated carbon (**AC**) amendment on root kin recognition responses of penoxsulam-resistant barnyardgrass (**RB**) in the presence of penoxsulam-susceptible barnyardgrass (**SB**) and normal barnyardgrass (**NB**). (**a**), Root biomass under root segregation; (**b**), Root biomass with and without soil activated carbon amendment; (**c**), Total root length with and without soil activated carbon amendment; (**d**), Total root area with and without soil activated carbon amendment. Values plotted are means plus/minus SE. Columns with the same letter within identical treatments are not significantly different at *p* < 0.05 according to ANOVA, followed by Tukey HSD tests.

**Figure 4:**
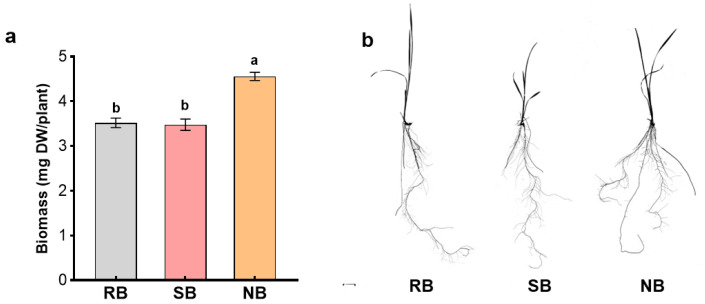
Root biomass (**a**) and morphology (**b**) of penoxsulam-resistant barnyardgrass (**RB**) in the root exudates of penoxsulam-susceptible barnyardgrass (**SB**) and normal barnyardgrass (**NB**). Values plotted are means plus/minus SE. Columns with the same letter are not significantly different at *p* < 0.05 according to ANOVA, followed by Tukey HSD tests.

When penoxsulam-resistant barnyardgrass and penoxsulam-susceptible barnyardgrass or normal barnyardgrass occurred together and interacted, root biomass of penoxsulam-resistant barnyardgrass were changed with and without root segregation. Root biomass consistently increased when grown with distantly related normal barnyardgrass under root contact and root segregation with 30 μm nylon mesh. However, the increased root biomass was not observed under root segregation with plastic film (Figure 3a). The plastic film blocked belowground physical, chemical, and biological interactions, limiting all interactions to aboveground. There were not significant differences on root biomass under root segregation with plastic film regardless of neighbor biotypes, indicating that the kin recognition responses relied on belowground interactions rather than aboveground interactions. Furthermore, the root measurements of penoxsulam-resistant barnyardgrass were significantly different in soil amended with and without activated carbon. Soil amended with activated carbon greatly changed root biomass, total root length, and total root area of penoxsulam-resistant barnyardgrass regardless of neighbor biotypes. In particular, significant changes in the root measurements of penoxsulam-resistant barnyardgrass induced by distantly related normal barnyardgrass were attenuated or terminated after soil activated carbon amendment (Figure 3b–d). When penoxsulam-resistant barnyardgrass was exposed to the root exudates of distantly related barnyardgrass biotype, the seedling produced larger root systems. However, root biomass and morphology were not significantly changed in the root exudates of closely related barnyardgrass biotype (Figure 4a,b). The results indicated the importance of belowground chemical interactions in mediating kin recognition among barnyardgrass biotypes.

### 2.3. The Role of (–)-Loliolide in Kin Recognition among Barnyardgrass Biotypes

**Figure 5:**
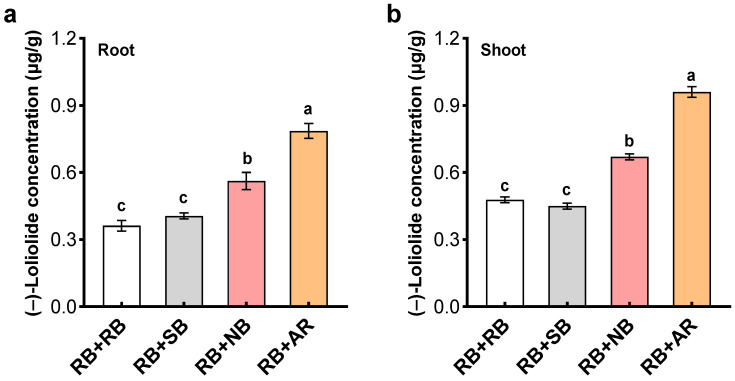
(–)-Loliolide levels in root (**a**) and shoot (**b**) of penoxsulam-resistant barnyardgrass (**RB**) in response to the presence of penoxsulam-susceptible barnyardgrass (**SB**) and normal barnyardgrass (**NB**), as well as allelopathic rice cultivar (**AR**). Values plotted are means plus/minus SE. Columns with the same letter are not significantly different at *p* < 0.05 according to ANOVA, followed by Tukey HSD tests.

Kin recognition indicates relatedness-mediated neighbor discrimination, mainly reducing intraspecific competition. While (–)-loliolide is a common signal for plant neighbor detection and competition [33,34]. This plant–plant signaling is reminiscent of the role of (–)-loliolide in kin recognition among barnyardgrass biotypes. Thus, we examined the level of (–)-loliolide in penoxsulam-resistant barnyardgrass in response to neighbors of differing relatedness. As expected, the concentration of (–)-loliolide in penoxsulam-resistant barnyardgrass significantly varied with neighbor relatedness (Figure 5). The more distant allelopathic rice cultivar led to the highest (–)-loliolide concentration in both root and shoot, followed by the distantly related barnyardgrass biotype. Penoxsulam-resistant barnyardgrass monocultures and those with closely related barnyardgrass biotype contained the lowest (–)-loliolide concentrations (Figure 5). This suggests that penoxsulam-resistant barnyardgrass may produce more (–)-loliolide in response to the presence of unrelated neighbors regardless of whether they are intraspecific or interspecific competitors, indicating (–)-loliolide’s role as a key component of penoxsulam-resistant barnyardgrass response to kinship interactions.

### 2.4. Rice-Barnyardgrass Allelopathic Interactions in Response to Kin Recognition among Barnyardgrass Biotypes

**Figure 6:**
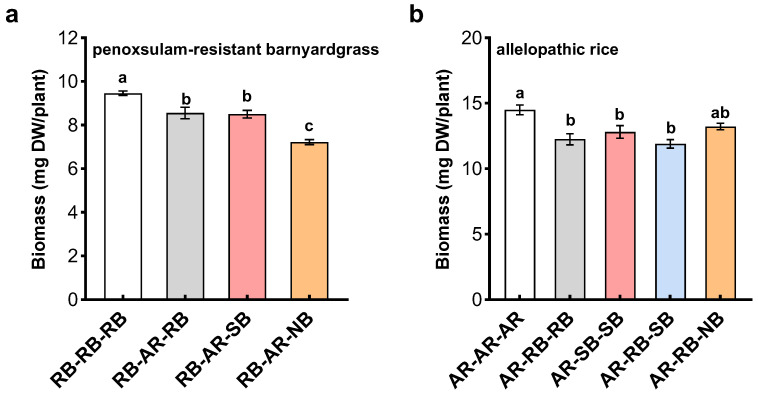
Effects of relatedness-mediated rice–barnyardgrass allelopathic interactions on biomass of penoxsulam-resistant barnyardgrass biotype (**a**) and allelopathic rice cultivar (**b**). **RB**, penoxsulam-resistant barnyardgrass biotype; **SB,** penoxsulam-susceptible barnyardgrass biotype; **NB**, normal barnyardgrass biotype; **AR**, allelopathic rice cultivar. Values plotted are means plus/minus SE. Columns with the same letter are not significantly different at *p* < 0.05 according to ANOVA, followed by Tukey HSD tests.

The presence of allelopathic rice cultivar significantly inhibited the growth of all barnyardgrass biotype mixtures, but the inhibition was dependent on the relatedness of mixed with barnyardgrass biotypes. There was more significant inhibition in penoxsulam-resistant barnyardgrass mixed with distantly related barnyardgrass biotype than in one mixed with closely related barnyardgrass biotype (Figure 6a). Similarly, relatedness-mediated inhibition occurred in the interference of barnyardgrass with allelopathic rice cultivar. Compared with rice monoculture, barnyardgrass biotype mixtures reduced rice biomass. However, significant reduction was observed in penoxsulam-resistant barnyardgrass mixed with the same and closely related biotypes rather than in one mixed with closely related barnyardgrass biotype (Figure 6b). These results showed that kin recognition among barnyardgrass biotypes affected the interactions between allelopathic rice and barnyardgrass. In particular, relatedness-mediated barnyardgrass biotypes cooperated resistance to inhibition of allelopathic rice cultivar, and enhanced the interference of barnyardgrass with allelopathic rice cultivar.

## 3. Discussion

Kin recognition in plants, as well as the evolutionary and ecological mechanisms underlying such recognition, has received a great deal of attention in the past decade [12,13,16,35]. However, there is still controversial issues in this fascinating area, particularly for the concept and definition of ‘kin’ in natural and managed ecosystems [36]. In natural ecosystems, ‘kin’ of wild species is limited to siblings or half-siblings and sometimes can be extended to plants sharing the same population [17,19,24]. In managed ecosystems, crops and weeds have undergone intense artificial selection and herbicide pressure, resulting in crop cultivars with genetic and morphological uniformity and the biotype of herbicide-resistant weeds [37]. Kin recognition at the cultivar or accession level has been observed in several crop species [18,21,22]. This study presents the first case of kin recognition at the herbicide-resistant weed biotype level.

Herbicide-resistant and -susceptible weeds simultaneously occur in cropping systems and evolve under the pressure of herbicides. The evolution of herbicide-resistant weeds is driven by the use of herbicides in large quantities. Most studies have focused on the incidence and gene mutation of herbicide-resistant weeds [26,27,37]. There is a lack of information on the interactions between herbicide-resistant and -susceptible biotypes or normal weeds. Such information is critical for understanding the coexistence, evolution, and mechanisms of weed biotypes and their consequences in cropping systems. From a system of penoxsulam-resistant and -susceptible barnyardgrass as well as normal barnyardgrass biotypes, we found the interactions among these biotypes. In particular, differential interactions were observed at varying relatedness level, indicating kin recognition of penoxsulam-resistant barnyardgrass at the biotype level. The direct evidence was that, when grown with closely related penoxsulam-susceptible barnyardgrass, there were (1) reduced root growth and distribution, lowering belowground competition and (2) advanced flowering and increased seed production, enhancing reproductive effectiveness. However, such kin recognition responses were not occurred when penoxsulam-resistant barnyardgrass was grown with a distantly related barnyardgrass biotype.

The root behavior is a key kin recognition response. Kin recognition can reduce competitive root systems, allowing greater allocation to reproduction [16,38,39]. Through window rhizobox and root segregation, this study clearly demonstrated the ability of penoxsulam-resistant barnyardgrass to respond to closely related penoxsulam-susceptible barnyardgrass, reducing competitive root systems. Penoxsulam-resistant barnyardgrass significantly increased their root growth and distribution in the presence of distantly related biotype but avoid the root growth and distribution in the presence of the same and closely related biotypes. Such relatedness-mediated root behavior to minimize root competition is key in creating more allocation to flowering and reproduction.

Flowering time is key to a plant reproductive strategy, and reproductive success depends on the flowering behavior of immediate conspecific neighbors [24,40,41]. Kin selection plays a role in the evolution of placentation and reproductive traits in flowering plants [42,43]. In particular, early flowering plants are favored by phenotypic selection on flowering phenology [44]. In the current study, penoxsulam-resistant barnyardgrass growing with closely related biotype accelerated flowering relative to those growing with distantly related biotype, providing a linkage to belowground root behavior and aboveground flowering and reproduction. In this manner, the penoxsulam-resistant barnyardgrass biotype may maximize their own growth to fit individual and population.

Kin recognition in plants involves both physical and chemical signals [45,46]. Most evidence suggests root exudates as the signal of relatedness [18,21,47]. In this study, we found consistent root measures of penoxsulam-resistant barnyardgrass regardless of neighbor biotypes in the presence of activated carbon. The tremendous adsorptive capacity of activated carbon for functional metabolites in soil alleviated root-secreted the signal of relatedness, resulting in kin recognition responses hard to occur. Furthermore, the exposure of penoxsulam-resistant barnyardgrass seedlings to the root exudates of distantly related biotype induced larger root systems than exposure of seedlings to the root exudates of their own or closely related penoxsulam-susceptible barnyardgrass biotype. These results agree with previous studies showing that root exudates mediate kin recognition in plants [18,21,47].

Cooperation arising from kin recognition allows cooperative behaviors to be directed preferentially toward kin that promotes the increase of offspring from related families, which is a mechanism to ensure the continuation and evolution of the population [48,49]. Kin recognition in plants can reduce intraspecific competition, allowing greater allocation to reproduction in kin groups or relatives mixtures. However, how to define the distinction between ‘kin’ and ‘non-kin’, actually quantifying the level of genetic relatedness is challenging particularly at the cultivar and biotype levels [36]. Several studies have used genetic distances by molecular approach to identify closely versus distantly related populations or cultivars [14,21,50]. In the current study, penoxsulam-resistant barnyardgrass significantly reduced (–)-loliolide production in the presence of closely related penoxsulam-susceptible barnyardgrass but increased production when growing with a distantly related barnyardgrass biotype and more distantly related interspecific allelopathic rice cultivar. In fact, kin recognition may allow plants to optimize competitive strategies, resulting in less intraspecific competition and more cooperation among plants [8]. Therefore, competition or cooperation are the most important, intraspecific traits between kin and non-kin. Relatedness allows plants to discriminate their neighboring collaborators (kin) or competitors (non-kin), either interspecifically or intraspecifically, and adjust their growth and competitiveness accordingly [14]. It appears from the results that the level of (–)-loliolide may indicate neighbor kinship, discriminating kin from non-kin and even differentiating interspecific competitors from conspecific competitors. (–)-Loliolide is a general signaling chemical for plant neighbor detection and plant competition, as well as other biotic and abiotic stressors [33,34]. (–)-Loliolide response to neighbor kinship may provide a chemical approach to quantify the level of genetic relatedness and define the distinction between ‘kin’ and ‘non-kin’ among crop cultivars and weed biotypes. Of course, for such (–)-loliolide-based interactions, the distinction between ‘kin’ and ‘non-kin’ needs to be verified in other plant systems.

Most kin recognition studies mainly focus on the occurrence and the extent to which plants express their cooperative behaviors towards conspecific neighbors based on the extent of genetic similarity between them [17,19,20,22,23]. In fact, plants often grow in mixtures of kin, non-kin conspecifics, and other species, and thus, intraspecific kin recognition and interspecific interactions may often occur simultaneously. Accordingly, an increasing number of studies have investigated how kin recognition mediates the context of cooperation between genetically related plants to fight against herbivores, to attract pollinators, and to compete with interspecific plants [14,24,50,51,52]. In particular, intraspecific kin recognition contributes to interspecific allelopathy in allelopathic rice interference with paddy weeds [14]. This study showed that penoxsulam-resistant barnyardgrass plants could modulate their root systems, flowering time and seed production in response to closely or distantly related biotypes. In this manner, penoxsulam-resistant barnyardgrass with the ability for kin recognition maximize inclusive fitness and stand performance. Importantly, genetically identical penoxsulam-resistant and -susceptible barnyardgrass biotypes synergistically interact to influence the action of allelopathic rice cultivar, providing evidence that kin recognition may also facilitate cooperation between genetically related plants to compete with interspecific intruders. The discovery of kin recognition in herbicide-resistant barnyardgrass at the biotype level, as well as a further understanding of its potential mechanism, may lead to evolutionary and ecological insights into herbicide-resistant weeds. A thorough understanding of intraspecific and interspecific interactions between crops and weeds in cropping systems will offer potential implications for applications and the development of sustainable agriculture strategies. 

## 4. Materials and Methods

### 4.1. Plant Materials, Soil, and Chemicals

An allelopathic rice (*Oryza sativa* L.) cultivar (Huagan-3) and two penoxsulam-resistant and -susceptible barnyardgrass (*Echinochloa crus-galli* L.) biotypes were used in this study. Huagan-3 is the first commercially approved allelopathic rice cultivar against paddy weeds in China [53]. The penoxsulam-resistant barnyardgrass seeds were originally collected from a rice field subjected to penoxsulam treatment for several consecutive years at Lujiang Experimental Station of Rice Research, Anhui province of East China (30.47° N, 117.38° E), which located on the side of the Yangtze River. Seed samples were taken for the experiments to identify and segregate penoxsulam-resistant and -susceptible individuals as described in a previous study [28]. Finally, the homozygous barnyardgrass seeds of penoxsulam-resistant and -susceptible biotypes were obtained by a 3-year experiment of continuous selection of penoxsulam application. The penoxsulam-resistant and -susceptible biotypes within a population and location possess the same genetic background, resulting in their relatedness. In addition, normal barnyardgrass seeds from a distant population without herbicide application were collected at Shenyang Experimental Station of Chinese Academy of Sciences, Liaoning Province of Northeast China (41°31′ N, 123°24′ E), which was distantly related biotype for both penoxsulam-resistant and -susceptible biotypes. Accordingly, penoxsulam-resistant barnyardgrass was selected as the focal biotype while penoxsulam-susceptible barnyardgrass was kin biotype and normal barnyardgrass was used as a more distant non-kin biotype.

Soil was collected randomly from the surface (0–10 cm) of a paddy field of Lujiang Experimental Station as described above. The soil is a typical fluvaquent, Etisol (US taxonomy) with pH 5.8, organic matter of 25.1 g/kg, total nitrogen of 1.6 g/kg, available phosphourus of 30.9 mg/kg, and available potassium of 60.4 mg/kg. Soil samples were air-dried, mixed, and then sieved (2 mm mesh) to remove plant tissues for a series of experiments as described below.

(–)-Loliolide, a putative root-secreted chemical signal involved in rice–barnyardgrass allelopathic interactions [30], was isolated and identified from root exudates using previously developed methods [33] and verified with its authentic standard obtained from Yuanye Biology Corporation (Shanghai, China). Other organic solvents and chemicals were purchased from China National Chemical Corporation (Beijing, China).

### 4.2. Pot-Culture Experiments of Mixed-Biotype Barnyardgrass

Two pot-culture experiments were conducted in a greenhouse at 20–30 °C night and daytime temperatures and 65–90% relative humidity maintained. Each experiment was conducted in a completely randomized design with three replicates for each treatment or control. The sterilized barnyardgrass seeds for each biotype were separately sown to Petri dishes (9 cm diameter) with moistened filter paper for pre-germination in a chamber set at a temperature of 28 °C.

The first experiment investigated the performance of penoxsulam-resistant barnyardgrass (focal biotype) in the presence of penoxsulam-susceptible barnyardgrass (kin biotype) or normal barnyardgrass (non-kin biotype) in a series of 15 (diameter) × 12 cm (height) plastic pots containing 2 kg of soil as described above. Four pregerminated seeds of penoxsulam-resistant barnyardgrass were spaced uniformly in the center of each pot while four seeds of penoxsulam-susceptible barnyardgrass or normal barnyardgrass were sown in the surrounding area. Pots with penoxsulam-resistant barnyardgrass monocultures in the same planting pattern served as controls. All pots were placed in the greenhouse, watered daily, and their positions randomized weekly. The penoxsulam-resistant barnyardgrass in 1/3 of pots were sampled at the seedling stage, and their shoots and roots were determined biomass and quantified for (–)-loliolide as described below (Section 4.6). In the remaining 2/3 of pots, flowering time and seed biomass of penoxsulam-resistant barnyardgrass were recorded at the flowering and mature stages, respectively.

A second experiment was run to evaluate the impact of root segregation on the performance of penoxsulam-resistant barnyardgrass in the presence of penoxsulam-susceptible barnyardgrass or normal barnyardgrass. A series of 11 cm (diameter) × 12 cm (height) plastic pots that contained a central cylinder (7.5 cm diameter, 12 cm height) where a barrier could be inserted were divided into three groups. The cylinders in the first group were not modified while the cylinders in the second and third group were covered with 30 μm nylon mesh or plastic film. The open cylinders were full contact. The 30 μm nylon mesh prevented penetration of root systems but allowed chemical and microbial interactions in the pots. The plastic film completely blocked root–soil interactions [33]. Four pre-germinated seeds of penoxsulam-resistant barnyardgrass were uniformly sown in the cylinder of each pot containing 800 g of soil; four pre-germinated seeds of penoxsulam-susceptible barnyardgrass or normal barnyardgrass were sown outside the cylinder of each pot. Monocultures of penoxsulam-resistant barnyardgrass (4:4) in a pot for each group with or without root segregation served as the controls. All the pots were placed in the greenhouse, watered daily, and randomized once a week. The seedlings of penoxsulam-resistant barnyardgrass were harvested after four weeks, and their biomass was measured.

### 4.3. Rhizobox Experiments of Mixed-Biotype Barnyardgrass

Root behavior of penoxsulam-resistant barnyardgrass in the presence of penoxsulam-susceptible barnyardgrass or normal barnyardgrass were determined using a window rhizobox method in a completely randomized design with three replications. The window rhizobox was made of a 20 cm (length) × 2 cm (width) × 30 cm (height) polyvinyl chloride box with a clear plexiglass sheet [54]. The penoxsulam-resistant barnyardgrass was grown in monoculture, or paired with penoxsulam-susceptible barnyardgrass or normal barnyardgrass in the window rhizoboxes containing 500 g of soil. The soils in half of the rhizoboxes were amended with 2% activated carbon. Each window rhizobox was vertically divided into two equal parts. A single penoxsulam-resistant barnyardgrass seed or an interacting barnyardgrass seed for each biotype was sown into each half. Monocultures of barnyardgrass for each biotype served as the controls. Window rhizoboxes were placed in racks and set to a 40° angle with the clear plexiglass sheet facing down and away from the light source in the greenhouse with 20–30 °C night and day temperatures and 65–90% relative humidity. The window rhizoboxes were opened after 4 weeks when root systems reached the horizontal or vertical margin of the rhizoboxes. The roots were scanned with an Epson Perfection V700 scanner (Seiko Epson, Nagano-ken, Japan) to yield a grey scale TIFF image [32]. The image was processed with the WinRHIZO system (Regent Instruments Inc., Quebec, Canada) to obtain three root measurements, including a size-related metric (total root length) and two measures of habitat occupancy (total root area and maximum root depth). Finally, the roots were freeze-dried for biomass determination.

### 4.4. Root Exudates Incubation

Root behavior of penoxsulam-resistant barnyardgrass in the root exudates of each biotype was evaluated with an incubation experiment. The root exudates of barnyardgrass for each biotype were collected hydroponically [28]. Sterilized and germinated seeds were sown in nursery seedling plates. Thirty barnyardgrass seedlings at the 2-leaf stage of each biotype were inserted into holes in a Styrofoam float and transplanted into a container (6 cm × 10 cm × 12 cm) containing 250 mL distilled water in a sterile growth chamber at 25 ± 1 °C with a 16 h light and 8 h dark photoperiod. After 7 days, the solution was filtered with sterile filter paper (GE Healthcare Whatman) and yielded the root exudates of each barnyardgrass biotype that were used for subsequent experiment.

A uniform penoxsulam-resistant barnyardgrass seedling at the 2-leaf stage was each transplanted into a sponge plug to stabilize the seedling that was then inserted into a series of transparent bottles with 100 mL root exudates of barnyardgrass for each biotype. All bottles in a completely randomized design with three replications were placed in a sterile environmental chamber at 28 °C with a 12 h photoperiod. After penoxsulam-resistant barnyardgrass seedlings in their own exudates reached the 3-leaf stage, all bottles were removed from the chamber. The seedling was each scanned to obtain a grey scale TIFF image (Figure 4b), and then, their root biomass was recorded.

### 4.5. Rice–Barnyardgrass Mixes-Species Experiment

The interactions between allelopathic rice cultivar and barnyardgrass biotypes were evaluated by mixes-species experiment. A series of plastic pots (12 cm diameter × 10 cm height) containing 1000 g of soil were used for the experiment. A total of eight pre-germinated seeds were sown into each pot. Four rice seeds were spaced uniformly in the central area (6 cm diameter) while four barnyardgrass seeds were sown in the outer circle in two biotypes cross manner. Monocultures of eight rice plants and four barnyardgrass plants within each biotype surrounding four rice plants served as the controls. The experiment was conducted in a completely randomized design with three replicates for treatment and control. All pots were placed in the greenhouse with 20–30 °C night and day temperatures and 65–90% relative humidity, watered daily, and randomized once a week. Both rice and barnyardgrass were harvested after four weeks, and their plants were separately freeze-dried for determining dry weight.

### 4.6. Quantification of (–)-Loliolide

Quantitative analysis of (–)-loliolide was performed by liquid extraction/solid-phase extraction followed by high-performance liquid chromatography (HPLC). Root and shoot of penoxsulam-resistant barnyardgrass were each freeze-dried and ground with liquid nitrogen. The resulting powder (250 mg) was extracted with 10 mL of a MeCN (acetonitrile)-H2O-HOAC mixture (90:9:1, *v*/*v*/*v*), vortexed for 5 min at 25 °C, and centrifuged at 2800× *g* for 10 min. The supernatant was each filtered with a 0.22 μm nylon syringe filter (Sterlitech, Kent, WA, USA). The filtrates were evaporated to dryness individually under vacuum. Dry residues were dissolved in 50% aqueous methanol and loaded onto reversed phase C_18_ Sep-Pak cartridges (Waters, Co., Milford, MA, USA), equilibrated with water, and eluted with MeOH. The MeOH fraction was concentrated with nitrogen gas to a final volume of 100 μL. The concentrated samples were subsequently subjected to a Waters 152 HPLC equipped with a C_18_ reverse-phase column (Hypersil 100 mm × 4.0 mm, 5 μm) and a diode array UV detector at 220 nm. Elution was performed with a mixture of 1 % acetic acid and MeOH (70:30, *v*/*v*) at a constant flow rate of 1.0 mL min^−1^ at 35 °C. The peak of (–)-loliolide was identified by its retention time (ca. 9.8 min) and coelution with an authentic standard (Yuanye Biology Co., Shanghai, China). (–)-Loliolide was quantified by regression analysis of the peak areas against standard concentrations.

### 4.7. Statistical Analysis

The normality and homogeneity of variances were verified in all statistical analyses. The data were analyzed using a one-way ANOVA followed by Tukey’s post hoc tests (HSD) to compare significant difference between treatments. All statistical procedures were carried out using SPSS 22.0 (IBM Corp., Armonk, NY, USA).

## Data Availability

All data supporting the findings of this research are available within the paper. Raw data used here can be obtained directly from the authors.
